# The Links Between Insecure Attachment to God, Divine Struggles, and Happiness and Depressive Symptoms Among Muslims and Jews in Israel

**DOI:** 10.1007/s10943-024-02055-y

**Published:** 2024-05-16

**Authors:** Tali Sasson Shoshan, Hagar Chaki-Binon, Hisham Abu-Raiya

**Affiliations:** 1 Ashkelon Academic College, Ashkelon, Israel; 2 Bob Shapell School of Social Work, Tel Aviv University, 69978 Tel Aviv, Israel

**Keywords:** Insecure attachment to God, Positive religious coping, Divine struggles, Depressive symptoms, Happiness

## Abstract

This investigation aimed to explore a theoretical model that examines the relationship between patterns of insecure attachment to God (i.e., anxious, avoidant), God-focused religious coping (i.e., divine struggles, positive religious coping), and mental health and well-being (i.e., happiness, depressive symptoms). The study's participants were 340 Israeli Jewish and Muslim individuals who completed electronic self-report questionnaires to assess the main variables of the study. The theoretical model was tested using Structural Equation Modeling. The analysis' findings indicated that there were no direct links between both patterns of insecure attachment to God and both happiness and depressive symptoms. Additionally, both anxious and avoidant attachment to God were found to be positively associated with divine struggles, and the latter mediated the relationship between both anxious and avoidant attachment to God and depressive symptoms. Furthermore, there were no significant associations between positive religious coping and any of the other variables in the study. Moreover, a comparative analysis revealed that the pattern of associations between the variables in the study was not dependent on gender or religious affiliation. Theoretical and practical implications of the findings are discussed.

## Introduction

The belief in a personal God, perceived as a secure attachment figure, constitutes a crucial component in the world's monotheistic religions (Kirkpatrick, 1998).^1^ In fact, the notions regarding God fulfill the four criteria of paternal attachment as defined by Bowlby (1969) and Ainsworth (1985): maintaining proximity with the attachment figure, perceiving the attachment figure as a secure foundation for exploratory conduct, considering the attachment figure as a secure sanctuary, and experiencing separation anxiety upon separation from the attachment figure (Kirkpatrick, 1999). Furthermore, various forms of religious encounters and behaviors, such as prayer, can be explicated in terms of the activation of the attachment system (Kirkpatrick, 1995).

Nevertheless, attachment to God diverges from attachment to human figures and is regarded by scholars as a symbolic attachment. It is, in essence, a result of humans' abilities to symbolize and mentalize. The development of these abilities enables individuals to engage with concealed figures like imaginary companions. However, while imaginary figures lose their significance for children over time and cease to be taken seriously by adults, God persists as a notable exception because adults in most world cultures regard the image of God with reverence and esteem. Additionally, God is perceived to possess distinctive powers that set Him apart from all other entities: He is omnipotent, omnipresent, and irreplaceable. These attributes contribute to His suitability as a symbolic attachment figure that believers strive to establish and/or actively maintain a sense of connection with (Grunqvist & Kirkpatrick, 2013).

Drawing upon this theoretical framework, Rowatt and Kirkpatrick (2002) identified three patterns of attachment to God, which mirror attachment to parental figures: secure (i.e., involvement with God perceived as warm and responsive), avoidant (i.e., involvement with a distant, impersonal, and unresponsive God), and anxious (i.e., involvement with an inconsistently responsive God). The latter two patterns fall within the realm of insecure attachment.

Although the concept of symbolic attachment to God is a relatively recent theoretical and empirical field, there is a growing body of empirical evidence linking attachment patterns to God with various aspects of health and psychological well-being. For instance, a secure attachment to God has been positively associated with better mental health (Pirutinsky et al., 2019), optimism, and self-esteem (e.g., Kent et al., 2018), lower levels of depression and anxiety (Kirkpatrick & Shaver, 1992), psychological distress and emotional problems (Bradshaw et al., 2010; Ellison et al., 2014), sense of loneliness (Kirkpatrick et al., 1999), and death anxiety (Gall & Bilodeau, 2020). On the other hand, anxious attachment to God has been negatively linked to self-acceptance and positive relationships (Homan, 2014) and positively associated with depression (Thauvoye et al., 2018), death anxiety, post-death depression, and death obsession (Mohammadzadeh & Oraki, 2020), whereas avoidant attachment to God has been positively associated with depression (Thauvoye et al., 2018) and negatively associated with perceived posttraumatic growth (Zeligman et al., 2020), empathic coping, and marital adjustment among spouses of women coping with breast cancer (Gall & Bilodeau, 2021).

The issue at hand is whether the connections attachment to God, mental health, and subjective well-being are direct or can be clarified through the mediation of other mechanisms. Nevertheless, there is a scarcity of literature concerning such mechanisms, particularly religious ones; only a few research studies have explored this potentiality. To illustrate, it was found that spiritual self-awareness acts as a mediator in the association between attachment to God and the ability to perceive suffering as a means of personal growth and connection with God (Bock et al., 2018). Additionally, self-compassion was found to mediate the link between patterns of insecure attachment to God and feelings of anxiety, depression, and life contentment (Homan, 2014), while fortitude mediated the relationship between the three patterns of attachment to God and mental health (Ghobary-Bonab & Khanjani, 2016). However, one plausible mediator between attachment to God and health and well-being, namely religious coping, has been largely overlooked. This study aims to address this gap in the existing literature.

### Religious Coping

Religious coping is defined as “*ways of understanding and dealing with negative life events that are related to the sacred*” (Pargament & Abu-Raiya, 2007). As its name indicates, religious coping is inherently derived from religious beliefs, practices, experiences, emotions, or relationships (Abu-Raiya & Pargament, 2015). Pargament et al. (1998) distinguished between two categories of religious coping: Positive religious coping and negative religious coping. Positive religious coping activities reflect a secure relationship with God, a belief that there is a greater meaning to be found, and a sense of spiritual connectedness with others, while negative religious coping activities reflect an ominous view of the world and a religious struggle to find and conserve significance in life. Factor analysis techniques indicate that positive and negative religious coping are higher-order constructs that describe a variety of more specific religious coping methods (Pargament et al., 2000).

The last three decades have witnessed a dramatic increase in studies examining the links between religious coping and the health and the well-being of the individual. These studies, which cover an array of populations and life stressors, have established significant associations between both positive and negative religious coping and indices of physical and mental health (for reviews, see Abu-Raiya & Pargament, 2015; Gall & Guirguis-Younger, 2013. For relevant meta-analyses, see Ano & Vasconcelles, 2005; Bockrath et al., 2021). By and large, empirical findings have linked positive religious coping to desirable physical and mental health indicators (e.g., higher life satisfaction and posttraumatic growth, and less depression and posttraumatic symptoms), and negative religious coping to undesirable ones (e.g., higher mortality rate and emotional distress, and lower happiness).

In this study, we focus on divine struggles as one form of a potentially problematic religious coping. In recent years, there has been a growing inclination to differentiate between religious struggles and negative religious coping; however, in this study we adhere to the conventional interchangeable application of these terms (Abu-Raiya & Pargament, 2015). Nevertheless, the term "struggles" is employed throughout the remainder of the manuscript due to its capacity to facilitate personal development in the aftermath of spiritual challenges and pressures. Divine struggles are one of 6 recently identified types of religious/spiritual struggles which occur when some aspect of religious and spiritual belief, practice or experience becomes a focus or a source of tension or internal conflict (Exline et al., 2014; Pargament & Exline, 2021). Divine struggles are unique in that they involve tensions or conflict centered on beliefs about God or a perceived relationship with God. For example, people can feel angry toward, punished, or abandoned by the divine (Exline et al., 2014). Research has shown that these struggles are common experiences of people dealing with stressful events (Grubbs & Exline, 2014). A significant of research has established divine struggles as a risk factor for poorer mental health and well-being (Cowden et al., 2024; see Exline 2013 for a review, and Bockrath et al., 2021 for a relevant meta-analysis).

**Fig. 1:**
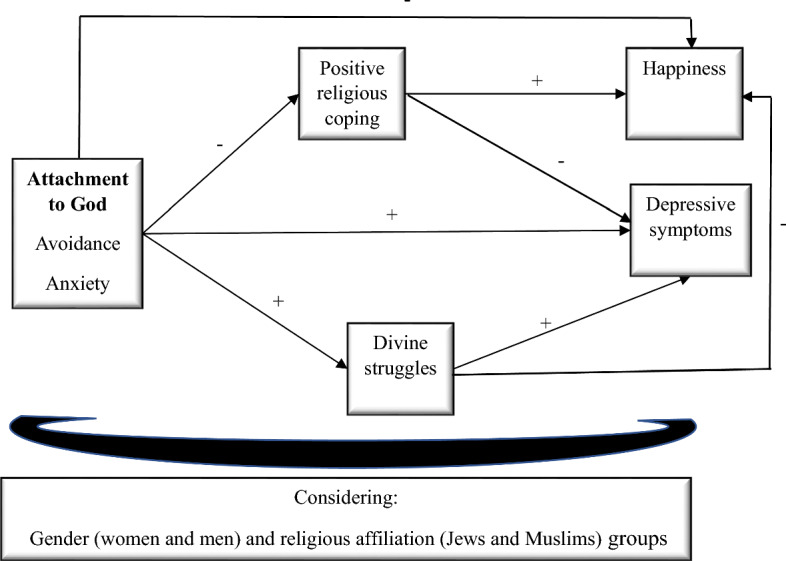
The theoretical model of the study

The purpose of the study is testing a model in which positive religious coping and divine struggles mediate the links between insecure attachment to God and both happiness and depressive symptoms. A visual presentation of the entire model is presented in Fig. 1.

We propose that that an exposure to stressful events would activate the insecure attachment to the God system. This activation, which is mostly a reactive, automatic process, might lead to the application of more active and conscious methods of coping, such as positive religious coping and divine struggles. These latter active and conscious processes are the ones that are more directly linked to happiness and depressive symptoms. Despite the theoretical plausibility of these assertions, only very few empirical studies have tested them empirically, with inconclusive conclusions. For instance, Davis et al. (2008) found that both positive and negative religious coping fully mediated the associations between anxious and avoidant attachment to God and forgiveness. Research by Kim (2015) and Kim et al. (2020) indicated that solely negative religious coping served as a mediator in the link between attachment to God and mental health as well as well-being outcomes. Conversely, Farrokhabadi and Ghobary Bonab (2018) identified that solely positive religious coping acted as a mediator in the relationship between attachment to God and marital conflicts.

A noteworthy study in this realm is the seminal research conducted by Belavich and Pargament (2002), who evaluated a comparable attachment to God-religious coping mediational model among adults with a loved one undergoing surgery. In providing theoretical rationale for the hypothesis that religious coping would mediate the impacts of attachment to God on outcomes, they posit that attachment to God serves as a broad "orienting system" variable that necessitates translation into more specific coping strategies during stressful periods. They contend that religious coping strategies embody particular thoughts, emotions, and behaviors that are closely and functionally linked to psychological consequences. Their findings offered some backing for negative religious coping as a mediator in the relationship between anxious attachment to God and outcomes.

We propose to scrutinize the model within the distinctive Israeli context. Specifically, the findings presented in this paper stem from a broader study that examined how religious elements are fused into coping with politically-induced stressors. This choice is deliberate; Israeli individuals, comprising both Jews and Muslims, endure persistent stress arising from conflicts and wars, which are characteristic of life in Israel (Michael, 2009). Within this context, a prevalent experience among Jewish and Muslim communities in Israel is a deep-seated sense of threat perceived by each group (Samuha, 2017). Moreover, religion occupies a central role in the lives of individuals in Israel, impacting both their daily routines and their capacity to manage psychological stressors. This assertion is corroborated by a mounting body of research on Muslim and Jewish populations in Israel (e.g., Abu-Raiya et al., 2015, 2016, 2017, 2020). These studies have unveiled the widespread utilization of religious coping mechanisms, encompassing both positive and negative aspects, with substantial implications for the well-being and health of community members. Additionally, these studies have underscored the disparities between these religious groups concerning the significance attributed to religion, God, and their perceived connection to the divine (Cooperman et al., 2016; Silverman et al., 2016).

Based on the literature review, we hypothesize that: (1) anxious and avoidant attachment to God will be negatively associated with happiness and positively associated with depressive symptoms, and; (2) positive religious coping and divine struggles will mediate the links between anxious and avoidant attachment to God and happiness and depressive symptoms.

## Method

### Procedure

The data for the current investigation was obtained from a broader study that was approved by the Ethics Committee of Tel Aviv University. Data collection was made by means of convenient sampling and snowball sampling techniques and was carried out in the months of June–July 2019. To be eligible for participation in the study, the individual needed to be an adult, and a native resident of Israel who declares on his/her belief in God. There was no restriction for participation in the study in terms of demographic data. The study's instruments were converted to electronic Hebrew and Arabic versions using the Qualtrics Software and were distributed through various relevant groups on the Facebook network along with an invitation to participate in the study and a link through which the questionnaire can be filled out on a secure website. Before participation, participants signed an informed consent. The collected data were automatically pulled into two SPSS files (data was collected in Arabic and Hebrew separately). Upon completion of data collection, the data were compiled into one SPSS file.

### Participants

The sample consisted of 340 participants (203 Jews and 121 Muslims) who live in different geographical areas of Israel. The relative proportion of Muslims in the sample was greater than their relative proportion in the population to ensure a meaningful comparative analysis. The socio-demographic characteristics of the study's participants and the differences between the religious affiliation groups (Jews and Muslims) in these characteristics are presented in Table 1.

**Table 1:** Participants' demographic characteristics

Variable	Whole sample		Jews		Muslims		*χ″* (δϕ)
	(*n* = 340)		(*n* = 203)		(*n* = 121)		
	*N*	%	*N*	%	*N*	%	
*Gender*							
Women	221	67.9	130	64.0	89	71.2	*χ″* (1) = 1.78
Men	109	32.1	73	36.0	36	28.8	
*Marital status*							
Married	196	57.6	130	64.0	66	52.8	
Single	98	28.8	46	22.7	49	39.2	*χ″* (4) = 14.24**
Separated or divorced	25	7.4	20	9.9	5	4.0	
widows and widowers	1	3.0	1	5.0	0	–	
Other	6	3.0	5	5.0	1	8.0	
*Religiousness							
Religious	81	24.6	44	21.7	37	29.6	*χ″* (2) = 38.34***
Traditional	142	43.2	68	33.5	73	58.4	
Secular	106	32.2	90	44.3	15	12.0	
*Monthly household income*							
Less than 3000 NIS	42	12.4	16	7.9	25	20.0	
Between 3000- 5000 NIS	53	15.6	22	10.8	30	24.0	*χ″* (3) = 33.15***
Between 5000–10000 NIS	126	37.1	76	37.4	50	40.0	
More than 10,000 NIS	104	41.4	84	41.4	20	16.0	
*Area of residence*							
Jerusalem	30	8.8	15	7.4	15	12.0	
North of Israel	117	34.4	22	10.8	93	74.4	*χ″* (4) = 160.09***
Tel Aviv/center/the triangle	78	22.9	66	32.5	12	9.6	
South of Israel and the Negev	78	22.9	75	36.9	3	2.4	
Other	26	2.6	25	4.4	0	–	
Age	*M(SD)*	*R*	*M(SD)*	*R*	*M(SD)*	*R*	*t (316)*
	36.9	70–18	39.2(11.5)	70–18	33.2(9.6)	62–18	4.76*

*Because a very small percentage of participants defined themselves as "Orthodox/very religious", the categories "religious" and "Orthodox/very religious" were grouped together into one category defined as "religious"

*p < .05; **p < .01; ***p < .001

Table 1 illustrates various demographic distinctions evident between individuals of the Jewish and Muslim faiths within the cohort under investigation. Notably, a greater percentage of Muslims were found to be single in comparison to their Jewish counterparts. Concerning religious adherence, a higher proportion of Muslims self-identified as traditional when juxtaposed with Jews, whereas a larger prevalence of secular individuals was noted among Jewish participants in contrast to Muslims. Furthermore, the data analysis revealed a higher average monthly household income for Jewish participants when compared to their Muslim counterparts. In terms of geographical distribution, a higher concentration of Jews resided in central Israel, while a greater number of Muslims were located in the northern regions of Israel in comparison to Jews. It is important to highlight that the majority of differences observed between Jewish and Muslim participants in the present sample align with documented distinctions within the wider Israeli populace (Central Bureau of Statistics, 2018; Weiss, 2018).

### Measures

#### Happiness

Happiness was assessed via the Subjective Happiness Scale, a 4-item scale developed by Lyubomirsky and Lepper (1999). For each item, participants were asked to circle the number that best characterizes them on a 7-point scale ranging from 1 (characterizing low levels of happiness) to 7 (characterizing high level of happiness). A sample item of this scale is "In general, I consider myself a very happy person." Higher scores on this scale indicate higher levels of happiness (*α* = 0.82).

#### Depressive Symptoms

Depressive symptoms were measured by a 10-item version of the Center for Epidemiological Studies Depression Scale (CES-D; Andresen et al., 1994). This version of the scale was translated from English into Hebrew and Arabic in a previous study (Abu-Raiya et al., 2017). Participants indicated how often they have experienced each symptom (e.g., “I was bothered by things that usually don’t bother me”) in the past week on a 4-point scale ranging from less than a day (1) to 5–7 days (4). Two items (e.g., “I was happy”) were reversed scored. Higher scores on this scale indicate greater depressive symptoms. Items were averaged (α = 0.87).

#### Attachment to God

Attachment to God was measured via the Attachment to God Inventory (AGI; Beck & McDonald, 2004). This scale is composed of 28 items, which reflect the perceived relationship with God. It contains 14 items which reflect avoidant attachment and 14 items which reflect anxious attachment. However, in the current investigation, two items were removed according to the recommendation of the questionnaire developers. Thus, the avoidant attachment subscale contained 14 items (e.g., “I just don’t feel a deep need to be close to God") and the anxious attachment subscale contained 12 items (e.g., “I worry a lot about my relationship with God”).

For each item, participants were asked to rate their degree of agreement on a 7-point scale ranging from (1) “strongly disagree” to (7) “strongly agree”. Seven of the items were reversed scored (e.g., “I am completely dependent on God for everything in my life”). Item scores were averaged in each subscale with higher scores indicating an anxious and/or avoidant attachment.

An exploratory factor analysis (EFA) was conducted on the items of this inventory. Based on this analysis, 7 items were removed: four of these removed items loaded similarly on both factors (i.e., “I often worry about whether God is pleased with me”; “I often feel angry with God for not responding to me when I want"; "Daily I discuss all of my problems and concerns with God"; " I am jealous when others feel God’s presence when I cannot”), whereases the remaining three had a low loading on its supposed factor (i.e., “It is uncommon for me to cry when sharing with God”; “I am uncomfortable being emotional in my communication with God”; “Even if I fail, I never question that God is pleased with me”). After removing these items, a good internal consistency (α = 0.86) was found for both anxious attachment and avoidant attachment to God.

#### God-Focused Religious Coping

Participants were prompted to think about a stressful political event they had experienced in the last three months and to complete the following two subscales.

#### Positive Religious Coping

Positive religious coping was assessed by the positive religious coping subscale of the Brief RCOPE (Pargament et al., 1998). This subscale is composed of 7 items, which reflect a generally secure relationship with the divine (Pargament et al., 2011) (e.g., “I did whatever I could and put the rest in God’s hands”). This subscale was translated from English into Hebrew and Arabic in a previous study (Abu-Raiya et al., 2017). Participants were asked to indicate the degree they used the coping strategy appearing in each item to cope with the exposure to the stressful political event on a 4-point scale ranging from (1) “I did or have been doing this a little bit” to (4) “I did or have been doing this a lot”. Higher scores on this subscale indicate greater positive religious coping. Item scores were averaged (α = 0.92).

#### Divine Struggles

Divine struggles were assessed by the 5-item “divine struggles” subscale of the Religious and Spiritual Struggles Scale (RSS; Exline et al., 2014). This subscale was translated from English into Hebrew and Arabic in previous studies (Abu-Raiya et al., 2015, 2016). Participants were asked to indicate to what extent they experienced the content in each item (e.g., “felt angry at God”) when they were exposed to the stressful political event, they were prompted to think of on a 4-point scale ranging from (1) “not at all” to (4) “very much”. Higher scores on this subscale indicate greater divine struggles. Item scores were averaged (α = 0.89).

## Results

### Descriptive Statistics

Table 2 displays descriptive statistics (i.e., mean, standard deviation, range) of the study’s main variables (i.e., anxious attachment to God, avoidant attachment to God, positive religious coping, divine struggles, happiness, and depressive symptoms) for both the total sample and for each of the two religious groups (Jews and Muslims).

**Table 2:** Descriptive statistics

	Whole sample			Jews				Muslims				
	*N*	*M*	*SD*	*R*	*N*	*M*	*SD*	*R*	*N*	*M*	*SD*	*R*
Anxious attachment to God	336	2.07	1.18	1–7	203	1.79	1.00	1–7	125	2.51	1.26	1–7
Avoidant attachment to God	335	3.66	1.52	1–7	203	4.16	1.52	1–7	125	2.89	1.18	1–7
Positive religious coping	338	2.41	.96	1–4	201	2.08	.85	1–4	125	2.93	.90	1–4
Divine struggles	335	1.35	.70	1–4	201	1.30	.68	1–4	122	1.44	.73	1–4
Happiness	339	5.16	1.10	1–7	203	5.24	1.09	1–7	124	5.05	1.09	1–7
Depressive symptoms	339	2.13	.68	1–4	203	2.11	.71	1–4	124	2.16	.63	1–4

Further analysis revealed that Muslims (*M* = 2.93, *SD* = 0.90) scored significantly higher than Jews (*M* = 2.08, *SD* = 0.85) on positive religious coping (*t* (324) = − 8.55, *p* < 0.001), whereas the two groups did not differ in their scores on divine struggles. With regard to patterns of attachment to God, Muslims (*M* = 2.51, *SD* = 1.26) scored significantly higher than Jews (*M* = 1.79, *SD* = 1) on anxious attachment to God [*t* (326) = -5.66, *p* < 0.01], whereas Jews (*M* = 4.16, *SD* = 1.52) scored significantly higher than Muslims (*M* = 2.89, *SD* = 1.18) on avoidant attachment to God [*t* (326) = 7.91, *p* < 0.01]. No differences were found between Jews and Muslims in their scores on both happiness and depressive symptoms.

### Correlational Analyses

Table 3 presents a correlation matrix including the study’s main variables. Anxious attachment to God positively correlated with both positive religious coping (*r* = 0.22, *p* < 0.01) and divine struggles (*r* = 0.53, *p* < 0.01). Conversely, avoidant attachment to God negatively correlated with positive religious coping (*r* = − 0.71, *p* < 0.01) and did not significantly correlate with divine struggles. None of the study main variables was related to happiness. However, positive links were found between depressive symptoms and both anxious attachment to God (*r* = 11, *p* < 0.01) and divine struggles (*r* = 0.17, *p* < 0.01).

**Table 3:** Correlation matrix

	Anxious attachment to God	Avoidant attachment to God	Positive religious coping	Divine struggles	Happiness	Depressive symptoms
Anxious attachment to God	–					
Avoidant attachment to God	−.21**	–				
Positive religious coping	.22**	−.71**	–			
Divine struggles	.53**	.02	.07	–		
Happiness	−.090	−.059	−.02	−.07	–	
Depressive symptoms	.11*	−.03	.06	.17**	−.33**	–

*p < .05; ** p < .01

### Findings of the Measurement Model

The measurement model was designed to examine the relationships between the latent variables and their observed indicators through the factor loading matrix (Heck, 2001). The following latent variables were included in the model: anxious attachment to God, avoidant attachment to God, positive religious coping, divine struggles, happiness and depressive symptoms. Since the measurement model is intended to exclusively examine how and to what extent the observed variables are related to the underlying latent variables (Byrne, 2013), at this stage the model did not specify the directions of the relationships between the variables and did not include restrictions on the relationships between these variables. The findings of the measurement model indicated a good fit to the data [χ^2^ (120, *N* = 340) = 236.405, p < 0.001, TLI = 0.963, CFI = 0.971, RMSEA = 0.053 (90% CI = 0.043, 0.063), SRMR = 0.046].

### Findings of the Structural Model

In the next step, we assessed the structural model in order to examine the relationships between the latent variables in the study and, accordingly, the manner in which certain latent variables directly or indirectly influence changes in the values of other latent variables in the model (Byrne, 2013).

**Fig. 2:**
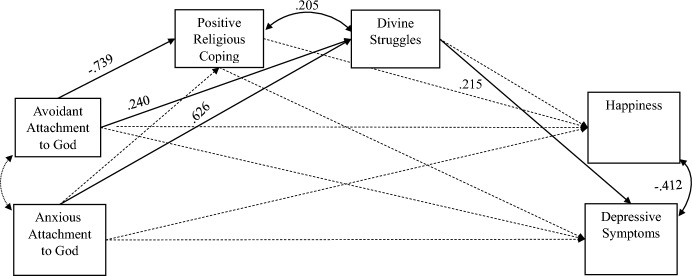
The structural model of the study among the total sample. *Note*. The solid lines in the model indicate significant paths at a significance level of *p* < .01. The dashed paths in the model indicate paths that were insignificant. *Note*. Goodness of fit indices: χ^2^ (196, N = 340) = 383.830, *p* < .001, TLI = .945, CFI = .956, RMSEA = .053 (90% CI = .045, .061), SRMR = .049

The structural model included the relationships between the latent variables arising from the study's theoretical model. Specifically, in this analysis, the following hypotheses were tested: (1) anxious and avoidant attachment to God will be negatively associated with happiness and positively associated with depressive symptoms, and; (2) positive religious coping and divine struggles will mediate the links between anxious and avoidant attachment to God and happiness and depressive symptoms. The demographic variables gender, marital status, religious affiliation and religiousness level were entered into the model as control variables. The analysis revealed a satisfactory fit to the data [χ^2^ (196, *N* = 340) = 383.830, p < 0.001, TLI = 0.945, CFI = 0.956, RMSEA = 0.053 (90% CI = 0.045, 0.061), SRMR = 0.049]. The path coefficients in this model are presented in Fig. 2.

No direct links were found between avoidant and anxious attachment to God and happiness and/or depressive symptoms. Thus, with the exception of divine struggles being positively related to depressive symptoms (*β* = 0.215, *p* < 0.01), the findings did not confirm the first hypothesis.

Regarding the patterns of attachment to God, avoidant attachment to God was found to be positively related to divine struggles (*β* = 0.240, *p* < 0.01) and negatively related to positive religious coping (*β* = − 0.739, *p* < 0.01). Anxious attachment to God, on the other hand, was positively related only to divine struggles (*β* = 0.626, *p* < 0.01) and was unrelated to positive religious coping.

The findings regarding the mediating effects revealed that no relationships were found between anxious and avoidant attachment to God and happiness through positive religious coping and divine struggles. However, a significant indirect effect was found in the positive relationship between anxious attachment to God and depressive symptoms through divine struggles (*β* = 0.135, *p* = 0.01) and a significant indirect relationship was found in the positive relationship between avoidant attachment to God and depressive symptoms through divine struggles (*β* = 0.052, *p* < 0.05). Hence, these findings partially confirmed the study's second hypothesis.

Further analyses were performed to test whether differences exist between Jews and Muslims, and between women and men, in the magnitude and direction of the model's simple relationships. Towards this end, an analysis of the general structural model was conducted both for the entire sample and for each of the four groups of religious (Jews and Muslims) and gender (women and men) affiliation separately. Then, a series of *Z* tests were conducted after Fisher's z transformation, which allows comparing different effect sizes (*r*) from different populations by converting them to a common index, *Zr*, which is distributed almost normally (see Rosenthal, 1991). Examining the direct and indirect links in the model did not yield significant differences between the religious affiliation and gender groups.

## Discussion

### Key Findings

Three key findings have emerged from the present investigation. The first finding, which is perhaps the most surprising, was that no direct link was found between both avoidant and anxious patterns of attachment to God and the outcome variables (i.e., happiness, depressive symptoms). This finding contradicts previous research suggesting that insecure attachment patterns to God have adverse effects on health and well-being (e.g., Bradshaw et al., 2019; Henderson & Kent, 2022; Mohammadzadeh & Oraki, 2020; Thauvoye et al., 2018; Zeligman et al., 2020). One possible explanation could have been that the relationship between attachment patterns to God and indicators of mental health and subjective well-being might be influenced by participants' self-reported level of religiousness, religious affiliation, or gender. However, further analyses conducted on the data did not provide support for these speculations. Thus, we concluded that anxious and avoidant attachment to God were not significant direct predictors of both happiness and depressive symptoms in Israeli society and required alternative approaches to shed light on this surprising finding.

One potential approach that could elucidate these findings is the socio-cultural context within which the study was conducted. Keller (2013) proposed that attachment theory should be redefined to become a more culturally sensitive conceptual framework. She argues that cultural contexts vary significantly in their working models and worldviews regarding autonomy, connection, and socialization goals. Therefore, attachment must be understood from a cultural and local perspective. While this assertion has primarily been applied to the attachment relationship between parents and children, it may be even more relevant to the attachment relationship with God, as religion is a concept that arises from a specific culture and time period (Guthrie, 1996). Following this line of thought, it can be argued that the characteristics and roles attributed to God take on different forms in various socio-cultural contexts. Furthermore, it is possible that belonging to a particular socio-cultural context has a stronger influence on the perceived image of God than religiousness, gender, and religious affiliation. Consequently, it is plausible that the binary question posed to participants at the outset of the present study—"Do you believe in God?"—does not fully capture the complexity of belief in God and should have been followed by a question addressing the nature and attributes of this God, such as "Which God do you believe in?".

Another potential explanation for the absence of correlations between attachment to God and happiness and depressive symptoms is that the Attachment to God Inventory (AGI, Beck & McDonald, 2004) utilized in this study may not fully capture the essence of attachment in the Israeli context. It is important to note that, based on an exploratory factor analysis (EFA), a significant number of items (specifically, 8) were removed from the original AGI due to their lack of conformity with the original factor structure. These findings lend support to the notion that studies conducted within the traditional attachment framework may not be entirely applicable to the study of attachment in diverse cultural contexts worldwide (Keller, 2013). This notion may be particularly relevant in the case of attachment to God, as some scholars argue that different groups may hold diverse perspectives on God. For instance, Voas and Day (2010) propose that although secular Christians may believe in God, He only plays a peripheral role in their worldview. He may serve as an explanatory entity, but not necessarily as one that actively intervenes in the social realm. In relation to the Israeli context specifically, it has been suggested that secular Jews in Israel perceive God differently from believers in monotheistic religions (Lahav, 2016). For the believing secular Jew, Jewish history may be viewed as a series of events that can be comprehended without invoking God's intervention (Liebman, 1997). Consequently, we advocate for a bottom-up approach that acknowledges the distinct ways in which individuals form attachments to God within their specific social and cultural contexts. Therefore, a more culturally-sensitive understanding of attachment to God is warranted.

The second notable finding of the present study is that the utilization of positive, God-focused religious coping strategies in response to exposure to stressful political events did not predict happiness or depressive symptoms, and thus did not mediate the relationships between patterns of attachment to God and happiness and depressive symptoms. To some extent, this finding is not unexpected, given that previous research on positive religious coping has yielded inconsistent results. While some studies have yielded findings similar to those of the current investigation (e.g., Abu-Raiya et al., 2020; McCleary-Gaddy & Miller, 2019), others have demonstrated that positive religious coping has direct and indirect positive effects on health and well-being (e.g., Abu-Raiya et al., 2011; Park et al., 2018). Furthermore, there is evidence to suggest that positive religious coping is positively associated with indicators of distress (e.g., Cornish et al., 2017; Khan et al., 2016). This empirical inconsistency raises the possibility that the manifestations and consequences of positive religious coping may vary across different socio-cultural contexts and in response to different stressors.

Another possible explanation for the absence of links between positive religious coping, happiness, and depressive symptoms maybe the "stress mobilization effect" (Pargament, 1997). Although the common assumption is that positive religious coping leads to a reduction in distress, it is possible that distress mobilizes the use of positive religious coping methods (Abu-Raiya & Pargament, 2015). If so, it is possible that the beneficial effects of positive religious coping with stressful political events in Israel are offset by the impact of the experienced distress that leads to the use of positive religious coping methods. Thus, the picture that is finally obtained is the absence of links between positive religious coping, happiness and depressive symptoms.

Further elucidation may be associated with the nature of the stressor analyzed in the present investigation. Certain prior research endeavors conducted in the sphere of exposure to stress of a political nature in Israel have failed to establish associations between this exposure and indicators of diminished mental health and well-being (e.g., Bitton & Laufer, 2018; Bleich et al., 2003). In an effort to explicate this seemingly unexpected finding, scholars have drawn attention to the phenomenon of habituation, whereby individuals who become progressively exposed to the stressful reality resulting from political-security threats gradually become accustomed to it and develop adaptive mechanisms. Consequently, and perhaps paradoxically, the level of stress decreases as these events recurs (Bleich et al., 2003). This habituation emerges as a source of resilience, particularly with regard to psychological well-being (Muldoon, 2013), and acts as a protective factor against the development of psychopathological symptoms associated with stress and trauma (Bitton & Laufer, 2018). Consequently, it is plausible to argue that, just as the prolonged duration of political stress in Israel has resulted in habituation, the utilization of positive religious coping in response to this exposure may have also undergone a process of habituation. Consequently, it is possible that a process of "double habituation" has occurred, wherein the extended period of turning to God without a fundamental change in reality has depleted the psychological benefit derived from turning to God.

The third principal finding of the present study was that divine struggles mediated the associations between both avoidant and anxious attachment to God and depressive symptoms. This study represents one of the first instances in which divine struggles have been established as an explanatory mechanism in the relationship between attachment to God and poorer mental health. It also lends credence to our theoretical framework positing that the activation of the attachment to God system, which is primarily a reactive and automatic process, may lead to mental health consequences through the utilization of more active and conscious coping strategies, such as divine struggles. Thus, it appears that during times of stress, perceiving God as emotionally unavailable (avoidant attachment) and/or as an abandoning figure (anxious attachment) may manifest as internal conflicts with God, or in other words, divine struggles. It is likely that the latter is what precipitates the "psychological damage".

### Other Notable Findings

In addition to the model-related findings of the study, several findings have emerged from the current investigation that are worth discussing. First, it was observed that Muslims exhibited significantly higher scores in positive religious coping compared to Jews. This finding aligns with previous research findings (e.g., Abu-Raiya et al., 2017, 2020) and is in line with the fundamental principles of the religious coping theory, which posit that religious coping behavior originates from an individual's overarching orienting system (Pargament, 1997) and becomes more apparent when individuals perceive their resources to be limited (Pargament & Abu-Raiya, 2007). Within the Israeli context, Muslims' religiousness level surpasses that of Jews (Central Bureau of Statistics, 2018), suggesting that religion likely plays a more central role in the belief system of Muslims than it does for Jews. Furthermore, Muslims in Israel constitute a marginalized minority group with the lowest socio-economic standing in society (Bental et al., 2017), which implies a scarcity of worldly resources compared to the Jewish population in Israel. Consequently, it is plausible that the higher prevalence of positive religious coping among Muslims, as opposed to Jews, can be attributed to the greater significance of religion in the lives of Muslims and the lack or limitation of other resources within the Muslim community. However, it was observed that levels of divine struggles were generally low across the entire sample, with no discernible difference between Jews and Muslims. Previous studies (Abu-Raiya et al., 2015, 2016) have suggested that religious struggles may not be prevalent within these religious groups due to socialization processes that promote stronger religious commitment, thereby reducing the likelihood of encountering divine struggles.

Second, the findings indicated that in terms of insecure attachment to God, Jews exhibited a significantly higher level of avoidant attachment in comparison to Muslims, whereas Muslims demonstrated notably higher levels of anxious attachment to God in contrast to Jews. These disparities may highlight the possibility that the relationship with God could encompass cultural dimensions. One cultural factor that could be pertinent to these results is the variations in religious devotion between Jews and Muslims residing in Israel. While a majority of Muslims in Israel identify themselves as adhering to traditional beliefs, the majority of Jews in Israel describe themselves as secular according to the Central Bureau of Statistics (2018). For a significant portion of Jews in Israel, the perception of God does not involve a belief in Him as the ultimate authority, but rather as a component of familial loyalty passed down through generations as suggested by Dinur (2017). Consequently, within this religious group, faith in God may be viewed as part of a logical framework that detaches the emotional bond with God, potentially leading to avoidant attachment. Conversely, individuals who are devoutly religious perceive God as a distinct and cohesive entity, with certain religious practices reinforcing their emotional connection with God. Among Muslims in Israel, the perception of God is characterized not only as a personal entity alongside humanity as articulated by Ali (2016), but also as the arbiter of human destiny according to Haj-Yahia (2004). Therefore, it is plausible that the emotional bond with God among this group could manifest an insecure attachment reflecting a desire for intimacy with God coupled with feelings of abandonment anxiety, indicative of an anxious attachment.

### Theoretical and Empirical Implications

The present study possesses two primary features that distinguish it from other studies. Firstly, it endeavors to merge two distinct theories within the field of psychology of religion, namely attachment to God theory and religious coping theory. Secondly, it investigates the correlation between various measures of religiousness and health and well-being in the Israeli context. Specifically, this study is the first to examine whether positive religious coping and divine struggles resulting from exposure to stressful political events mediate the relationships between attachment to God, happiness, and depressive symptoms. From a theoretical perspective, the current findings underscore the significance of considering the socio-cultural context when examining different forms of religiousness and their associations with various aspects of health and well-being. The absence of direct connections between attachment to God, happiness, and depressive symptoms, as observed in this study, contradicts previous studies primarily conducted in Western cultures and among Christian populations, thus strongly suggesting that the impact of attachment to God on health and well-being may be contingent upon the socio-cultural context. These findings also raise the possibility that divine struggles may, to some extent, be a manifestation of insecure attachment to God or an active mechanism triggered in response to such insecurity. Furthermore, these findings cast doubt on the uniform "positivity" of positive religious coping. It is plausible that a more specific approach to this phenomenon, one that takes into account the specific stressor, indicators of health and well-being, and the research context, is warranted.

### Limitations and Future Directions

The results of this study should be interpreted in light of the following limitations. First, the sample was relatively small and was nonrandomly selected. Also, the participants answered the study questionnaires online, so access to computing devices and basic computing knowledge were required to participate in the study. This fact might have excluded individuals from some sectors of the Israeli population from participating in the study. These facts limit the generalizability of the findings to the larger Israeli population. Future studies should attempt to replicate and generalize these findings among a representative sample of Israeli, Jews and Muslims, adults. Second, the study design was cross-sectional so that causality cannot be inferred from these findings. Longitudinal studies are needed to better establish the causal connections between the variables included in the study's model. Finally, it is possible that self-reporting on religious aspects and in particular, attachment to God and God-focused religious coping, is subject to biases arising from "theological desirability" (Abu-Raiya, 2017), that is, the motivation to present religion in a positive light and avoid reporting on feelings, thoughts and actions that depict one's own religion on in a negative manner. In a similar vein, reporting on the outcome variables (happiness and depressive symptoms) is also subject to bias arising from self-reporting, namely social desirability-the motivation to present oneself positively. However, self-report would seem to be the most appropriate way to assess attachment to God and religious coping methods since they are experienced, for the most part, internally. Finally, the request for participants to answer inquiries concerning a recent stressful political event might have overlooked how individuals cope with stress more broadly in relation to factors like health and relationships. It is possible that numerous individuals were not distressed by a recent political event, hence not seeking divine intervention. Consequently, this could have diluted their responses to questions about positive religious coping, impacting their level of happiness or depression.

## Conclusion

This investigation aimed to explore a theoretical model that examines the relationship between patterns of insecure attachment to God (i.e., anxious, avoidant), God-focused religious coping (i.e., divine struggles, positive religious coping), and mental health and well-being (i.e., happiness, depressive symptoms). Its findings indicated that there were no direct links between both patterns of insecure attachment to God and both happiness and depressive symptoms. Additionally, both anxious and avoidant attachment to God were found to be positively associated with divine struggles, and the latter mediated the relationship between both anxious and avoidant attachment to God and depressive symptoms. Furthermore, there were no significant associations between positive religious coping and any of the other variables in the study. Moreover, a comparative analysis revealed that the pattern of associations between the variables in the study was not dependent on gender or religious affiliation.

The study’s findings emphasize the importance of considering the socio-cultural context in the analysis of various manifestations of religiousness and their correlations with different aspects of health and well-being. The lack of direct links noted in this research between one's attachment to God, feelings of happiness, and symptoms of depression contradicts earlier studies primarily carried out in Western societies and among Christian communities, strongly indicating that the influence of one's attachment to God on health and well-being might rely on the socio-cultural setting. These findings also suggest the potential that divine struggle could be partly a result of an insecure attachment to God or a responsive mechanism triggered by such insecurity. Moreover, these results question the consistent "positive" nature of positive religious coping. It is conceivable that a more precise approach to this concept, one that considers the specific stressor, health and well-being indicators, and the research environment, is justified.

## Footnotes

1 The linguistic framework utilized in this study is monotheistic in nature as it examines two demographic groups who follow monotheistic faiths, specifically Judaism and Islam.
